# Factors affecting length of hospital stay in stroke survivors in South Africa: A call for a stroke unit

**DOI:** 10.4102/ajod.v11i0.1065

**Published:** 2022-12-12

**Authors:** Stephanie C. Pillay, Roxann Redant, Nadia Umuneza, Azra Hoosen, Fiona Breytenbach, Sameera Haffejee, Zvifadzo Matsena-Zingoni, Kganetso Sekome

**Affiliations:** 1 Department of Speech Therapy and Audiology, Chris Hani Baragwanath Academic Hospital, Johannesburg, South Africa; 2 Department of Occupational Therapy, Chris Hani Baragwanath Academic Hospital, Johannesburg, South Africa; 3 Department of Physiotherapy, Chris Hani Baragwanath Academic Hospital, Johannesburg, South Africa; 4 Department of Occupational Therapy, University of the Witwatersrand, Johannesburg, South Africa; 5 Division of Epidemiology and Biostatistics, University of the Witwatersrand, Johannesburg, South Africa; 6 Department of Physiotherapy, University of the Witwatersrand, Johannesburg, South Africa

**Keywords:** stroke, rehabilitation, length of stay, stroke unit, South Africa

## Abstract

**Background:**

Stroke in Africa is a growing and neglected crisis with the incidence more than doubling in low- to middle-income countries in the last four decades. Despite this growing threat, implementation of stroke models of care in hospitals is lacking. Stroke units as a model of care have been shown to decrease mortality, reduce length of hospital stay (LOS) and improve outcomes in stroke survivors.

**Objectives:**

To determine the profile of stroke survivors and identify factors contributing to LOS at Chris Hani Baragwanath Academic Hospital (CHBAH) in South Africa to support stroke unit implementation.

**Method:**

This study involved a retrospective record review of stroke survivors admitted to CHBAH between September 2018 and May 2019. Factors associated with LOS were determined using linear regression models; univariate and multiple regression models were fitted.

**Results:**

A total of 567 participants’ data were included. Overall, 51.85% of the participants required services from all rehabilitation disciplines. The median LOS was 9 days (interquartile ranges [IQR]: 5–11 days) with each discipline providing an average of six sessions. Participants who were referred to the rehabilitation team 3 days after admission to hospital stayed 6 days longer compared with those participants who were referred earlier (*p* < 0.001).

**Conclusion:**

Delayed referral to the rehabilitation team resulted in increased LOS. This study supports the need for dedicated stroke units to decrease hospital LOS and improve patients’ outcomes by ensuring early, well-coordinated rehabilitation intervention and discharge planning.

**Contribution:**

The study highlights the urgency for re-evaluation of stroke care infrastructure within Gauteng to streamline and provide accessible stroke models of care.

## Introduction

Stroke in Africa is a growing burden and neglected crisis (Owolabi et al. 2015). The majority of the global stroke burden is in low- to middle-income countries with stroke incidence more than doubling in these countries in the last four decades (Johnson et al. 2016). In South Africa, strokes are estimated to be responsible for 25 000 deaths annually and 95 000 years lived with disability (Maredza, Bertram & Tollman 2015). The poor health of the South African population is often attributed to the quadruple disease burden the country faces (Pillay-Van Wyk et al. 2016). The increase in various comorbidities and non-communicable diseases as a risk factor for stroke has further contributed towards an increased prevalence of strokes (Mudzi, Stewart & Musenge 2012; Owolabi et al. 2015). Currently in South Africa’s public healthcare system, most stroke survivors are managed as part of a general medical service without dedicated beds assigned to stroke care (Bryer et al. 2010). Despite the growing threat to the healthcare system, implementation of stroke models of care within hospitals is lacking (Bryer et al. 2010; Gouda et al. 2019; South African Contextualised Stroke Rehabilitation Guideline [SACSRG] 2019).

The implementation of stroke units is widely recognised as the most effective solution to managing the burden of acute strokes (Bryer et al. 2010; Gittins et al. 2020; Grimmer et al. 2019; SACSRG 2019). Literature defines a stroke unit as an environment in which specialised and multidisciplinary stroke teams deliver stroke care in a dedicated ward or unit (Canadian Stroke Strategy 2009; Pierot et al. 2018). Treatment in a stroke unit has been found to decrease mortality, reduce length of hospital stay (LOS) and improve patient outcomes because intervention is early and well coordinated (Bryer et al. 2010; SACSRG 2019; Viljoen 2016; Zhu et al. 2009). Moreover, stroke unit implementation does not come at a high cost to resource-constrained facilities. No additional staffing is required, and it can be easily established by allocating a ward dedicated to the acute care of stroke survivors (Adeloye 2014). With an existing trained multidisciplinary team and established medical wards, the stroke unit model of care is a potentially feasible model to be implemented in public hospitals in South Africa. Considering the enormous economic burden that strokes place on healthcare systems, stroke rehabilitation services need to become a strategic priority for the public health sector (Bryer et al. 2010; Owolabi et al. 2015).

There is limited data on the stroke unit model of care in South Africa and the specific understanding of stroke patterns, determinants and outcomes (Grimmer et al. 2019; Owolabi et al. 2015). This study will therefore help provide more contextually relevant data to South Africa. This research aimed to explore the need for a stroke unit based on the profile of acute stroke survivors referred for rehabilitation at the largest central hospital in South Africa. The primary objectives of this study were to understand the profile of the acute stroke population, determine the factors affecting LOS and outline the current pathway of stroke survivors.

## Methodology

### Study design

A retrospective record review was conducted using patient records retrieved for stroke survivors admitted as inpatients at Chris Hani Baragwanath Academic Hospital (CHBAH) who received Speech Therapy (ST), Occupational Therapy (OT) and Physiotherapy (PT) services between September 2018 and May 2019.

### Study participants

**FIGURE 1:**
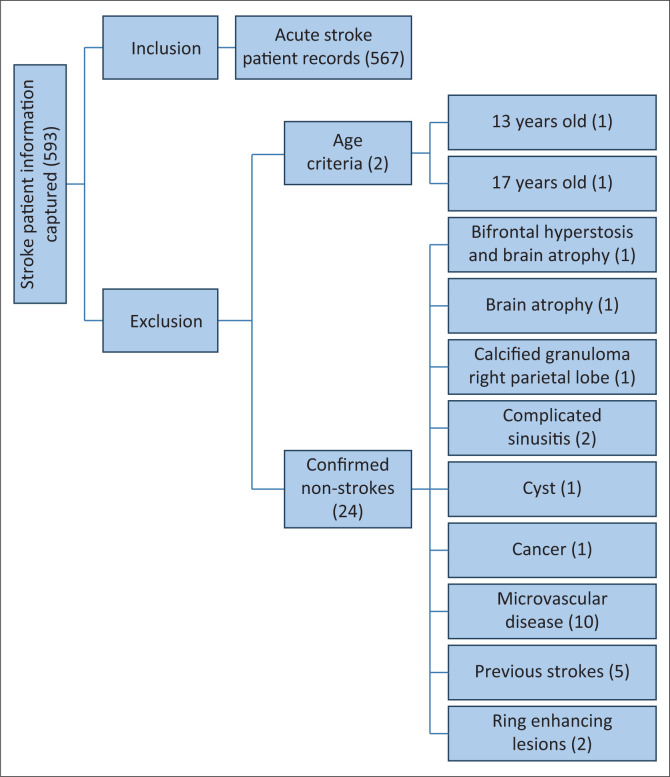
Flow diagram of included and excluded patient records.

The rehabilitation team retrieved records of 593 patients referred for rehabilitation within the study period. The exclusion criteria included: patients admitted for medical reasons other than acute strokes, patients who were admitted and discharged prior to referral to the rehabilitation team, records of patients who had demised prior to referral to the rehabilitation team and patients below the age of 18 years old. The study focused on analysis of the 567 patient records that met the inclusion criteria. Figure 1 demonstrates the inclusion and exclusion criteria.

### Study setting

Data were retrieved from a public healthcare institution, CHBAH, in South Africa. CHBAH is the third largest hospital in the world, accommodating approximately 3400 beds.

### Data extraction and instrument

The rehabilitation team extracted data from participants’ medical records using a manual data collection tool. The variables collected included gender, age, date of admission, comorbidities, type of stroke, area of lesion, date of referral to rehabilitation team, date of initial contact and the patient’s management outcome. The LOS was measured from the date of admission to the date of discharge from hospital or rehabilitation services as well as death. The data were transferred to an electronic Excel Sheet and verified against original hospital records, electronic hospital records, the Picture Archiving and Communication System and the National Health Laboratory Service electronic database.

### Data analysis

The management, cleaning, coding and analysis of data were performed using STATA version 16.1. Continuous variables such as age were summarised using mean and standard deviation (SD) as they were normally distributed. Skewed variables such as the LOS were summarised using medians and interquartile ranges (IQR). All categorical variables (gender, stroke type, stroke site, etc.) were compiled using frequencies and proportions. The factors associated with LOS were determined using the linear regression model, and both univariate and multiple regression models were fitted. Significance was based on 5% across all analyses.

### Ethical considerations

The data used in this study were anonymised using unique identifiers per participant. Permission to use the participants’ hospital records was granted by the Chief Executive Officer and Heads of Departments at CHBAH. This study obtained ethical clearance from the Human Research Ethics Committee of the University of the Witwatersrand (Ethical clearance number: NO. M190744).

## Results

A total of 567 participants were included in the study. Table 1 describes the demographic characteristics of the participants. The participants’ ages ranged from 18 to 99 years old, with a mean age of 61 (SD 15.8) years old. Majority of the participants (44.8%) fell in the 59–78 years age group. Females accounted for 55% of the participants and 78% of the participants had an ischaemic stroke. There were 82.4% participants with an underlying condition. Among all participants, 69.8% were hypertensive 20.1% had diabetes mellitus, 18.9% were HIV positive and 5.3% had tuberculosis (TB) infection. However, many participants were not tested for HIV or TB, with the status being unknown for 49% and 70.4% of participants, respectively.

**TABLE 1:** Demographic profile of stroke survivors admitted to Chris Hani Baragwanath Academic Hospital between September 2018 and May 2019 (*n* = 567).

Variable	Categories	Frequencies	%
Age (in years)	18–38	49	8.6
	39–58	177	31.2
	59–78	254	44.8†
	79–100	81	14.3
	Missing	6	1.1
Gender	Female	312	55.0†
	Male	255	45.0
Type of stroke	Ischaemic	442	78.0†
	Haemorrhagic	62	10.9
	Unknown	63	11.1
HIV status	Negative	182	32.1
	Positive	107	18.9
	Unknown	278	49.0†
Tuberculosis (TB)	Negative	138	24.3
	Positive	30	5.3
	Unknown	399	70.4†
Hypertension	Positive	396	69.8†
	Negative	95	16.8
	Unknown	76	13.4
Diabetes mellitus	Negative	288	50.8†
	Positive	114	20.1
	Unknown	165	29.1

† , values highlight the largest percentage.

**FIGURE 2:**
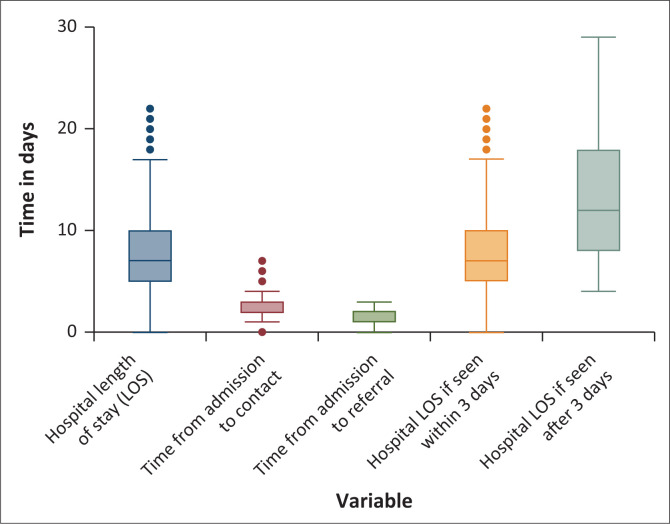
Box and whisker plots for various length of hospital stay intervals evaluated for the stroke survivors.

The LOS of participants in the study ranged between 0 and 76 days. The median number of days spent at CHBAH was 9 days with an IQR of 6–14 days. The median LOS of the participants referred to and seen by the rehabilitation team within 3 days of admission was 8 days (IQR: 5–11 days), while the median LOS of the participants referred and seen by the rehabilitation team after 3 days of admission was 13 days (IQR: 8–20 days). The median LOS time difference between those seen within 3 days and those seen after 3 days was significant (*p* < 0.0001) as shown by the box and whisker plot in Figure 2.

The LOS was stratified by different baseline characteristics to identify any sub-population variations (Table 2). There was a significant median difference in the LOS between age groups (*p* = 0.0021), HIV status categories (*p* = 0.0259), TB status categories (*p* = 0.0124) and hypertension status groups (*p* = 0.0056). There was a negative correlation between age at admission in years and LOS in days of 15.7%, which was fairly weak; however, the linear relationship was statistically significant (*p* = 0.0002).

**TABLE 2:** To determine the factors contributing to length of hospital stay of participants using univariate and multiple linear regression.

Variable	Categories	Univariate analysis			Multiple analysis		
		Coefficient	95%CI	*p*	Coefficient	95%CI	*p*
Age groups (in years)	18–38	base					
	39–58	−4.764	−7.552 – −1.977	0.001	-	-	-
	59–78	−5.333	−8.024 – −2.641	< 0.001	-	-	-
	79–100	−6.329	−9.451 – −3.208	< 0.001	-	-	-
Age in years	-	−0.088	−0.134 – −0.042	< 0.001	-	-	-
Gender	Female	Base			Base		
	Male	1.571	0.106 – 3.035	0.036	1.061	−0.363 – 2.484	0.144
Type of stroke	Haemorrhagic	Base	-	-	-	-	-
	Ischaemic	−0.05	−2.331 – 2.331	0.967	-	-	-
	Unknown	−1.883	−5.001 – 1.236	0.236	-	-	-
HIV status	Negative	Base	-	-	-	-	-
	Positive	2.809	0.699 – 4.919	0.009	-	-	-
	Unknown	0.427	−1.225 – 2.079	0.612	-	-	-
Tuberculosis (TB)	Negative	Base	-	-	Base	-	-
	Positive	4.147	0.629 – 7.664	0.021	2.017	−1.441 – 5.475	0.252
	Unknown	1.945	0.239 – 3.652	0.026	1.487	−0267 – 3.242	0.096
Hypertension	Negative	Base	-	-	Base	-	-
	Positive	−3.026	−5.013 – −1.039	0.003	−2.384	−4.412 – −3.55	0.021
	Unknown	−0.835	−3.495 – 1.824	0.538	−1.149	−4.306 – 2.007	0.475
Diabetes mellitus	Negative	Base	-	-	base	-	-
	Positive	−2.525	−4.435 – −6.156	0.01	−1.755	−3.652 – 0.141	0.07
	Unknown	−0.157	−1.847 – 1.532	0.855	−0.162	−2.286 – 1.961	0.881
Number of rehabilitation team members in contact with patient	1	Base	-	-	Base	-	-
	2	0.907	−1.492 – 3.308	0.458	1.913	−0.399 – 4.225	0.105
	3	3.501	1.201 – 5.802	0.003	4.692	2.476 – 6.908	< 0.001
Referral categories	Within 3 days	Base	-	-	Base	-	-
	After 3 days	5.762	4.163 – 7.361	< 0.001	5.878	4.313 – 7.443	< 0.001

CI, confidence interval.

To determine the factors associated with LOS, linear regression was performed. There was a dose–response relationship between age groups and LOS. The older the participant the less likely they were to stay longer in the hospital. A one-year increase in participants’ age decreased the LOS by 0.1 days. Male participants had an increased LOS of 2 days compared with females (*p* = 0.036). Participants with HIV infection stayed 3 more days in the hospital compared with HIV negative participants (*p* = 0.009). Study participants with TB infection stayed 4 more days in the hospital compared with TB negative participants (*p* = 0.021). Study participants with hypertension stayed 3 fewer days compared with those who were not hypertensive and those with diabetes mellitus stayed 3 fewer days compared with those without diabetes mellitus. The stroke survivors who required rehabilitation from all three professions stayed 4 more days compared with those who required rehabilitation from one profession (*p* < 0.003). In the adjusted linear regression, gender and having TB infection were no longer significantly associated with LOS. However, factors such as hypertension, diabetes mellitus and referral to the rehabilitation team still remained statistically significant.

Majority of the participants were first referred to OT followed by PT and lastly ST. Cross tabulations of the referral and contact patterns by profession showed an 84.1% agreement that the participants who were first referred to a specific profession had their first contact with that particular profession. Of all the participants reviewed, 51.9% required rehabilitation services from all rehabilitation team members. Out of the total number of participants, 89.4% required PT, 82.7% required OT and 67.2% required ST. The data further indicate that the rehabilitation team was able to attend to 53.4% of referrals within 24 h. Each discipline had an average of six sessions with a stroke survivor prior to hospital discharge. Majority of the participants were referred and seen by the rehabilitation team within 3 days of admission (*n* = 391, 71.7%) while 28.26% (*n* = 154) were referred and seen by the rehabilitation team after 3 days.

Table 3 further outlines the outcome of participants after inpatient care for each discipline. Most of the participants (60.7%) were referred to a local clinic or other hospital to continue stroke rehabilitation. The second most common outcome was death with 19.8% of the participants passing away while in hospital. On average, 7.2% of participants were discharged by the rehabilitation team; this occurs when a patient has improved and no longer requires therapy or if the patient’s medical condition has deteriorated or plateaued. Among the 111 participants who died, most of them were males (54%), had an ischaemic type of stroke (75.6%) and 58.5% were in contact with all the three rehabilitation professions and received care within 3 days from admission. They had a median LOS of 7 days (IQR: 6–10 days). The data indicates that only 2.1% of all participants in the study were transferred to a specialised rehabilitation hospital to continue intensive stroke rehabilitation.

**TABLE 3:** Determining the care pathway and participant outcomes.

Variable	Categories	Frequencies	%
Number of the professionals who provided patient care	Required rehabilitation from all 3 rehabilitation team members	**294**	**51.9**
	Required rehabilitation from two rehabilitation team members	202	35.6
	Required rehabilitation from one rehabilitation team member	71	12.5
Outcomes following discharge or end of service	Referred for follow-up rehabilitation	385†	67.9†
	Passed away	111	19.8
	Discharged from rehab services	41	7.2
	Discharged from the hospital without a follow-up	16	2.8
	Referred to a long-term rehab hospital	12	2.1
	Missing information	2	0.4

† , values highlight the largest frequency and percentage.

## Discussion

The successful management of a stroke begins with the recognition of stroke as an emergency (Bryer et al. 2010). As a central level hospital with a high admission of strokes, the management of stroke survivors should be a strategic priority in improving patient outcomes, reducing the cost to healthcare and the burden of strokes on hospital beds. Literature indicates that stroke care can be improved by implementing evidence-based stroke care pathways (Andrew et al. 2019; Langhorne, Pollock & Stroke Unit Trialists’ Collaboration 2002). Solutions to managing the high incidence of strokes in low- and middle-income countries need to be generated (Johnson et al. 2016). One solution to managing the high burden of strokes includes the implementation of a stroke unit (SACSRG 2019). The discussion aims to review the demographic profile of the participants, the factors that impacted their LOS and the care pathway they followed through the hospital setting in order to demonstrate how a stroke unit can improve their overall care while reducing hospital costs and LOS.

The 75.2% occurrence of ischaemic stroke in this study is in line with existing stroke literature that indicates that ischaemic strokes are more common (Boehme, Esenwa & Elkind 2017; Campbell et al. 2019; Girijala, Sohrabji & Bush 2017). The data demonstrated a higher prevalence of strokes in females. This is similar to findings of an existing South African study that highlighted a higher prevalence of strokes among females (Ferris & Naicker 2020). Despite age-specific stroke rates being higher in males, females are more at risk of having more stroke events that they are less likely to recover from because of their longer life expectancy (Girijala et al. 2017; Jacobs & Ellis 2021; Reeves et al. 2008). These current findings are also comparable to a study conducted at CHBAH, which found ischaemic strokes more prevalent in women than men (Mudzi et al. 2012).

Strokes in Africa occur at a younger age and are more deadly (Johnson et al. 2016). The average age of the study participants was identical to a previous study in a similar context, which found the average age of stroke survivors to be 59.8 years (Ferris & Naicker 2020). The age profile of stroke survivors is important because strokes in younger people may result in a greater loss of self-worth and socioeconomic productivity (Akinyemi et al. 2021). In this study, age was found to be statistically significant to the LOS and demonstrated a negative correlation pattern (i.e. the older the stroke survivor the shorter the LOS). The findings contrast a number of studies that found the older the stroke survivor the longer the LOS (Peltola & Euro 2012; Saxena & Prasad 2016). Available literature on this correlation was largely based on developed nations and reasons for the deviation in this study is likely multifactorial and requires further investigation.

Sixty nine percent of study participants presented with hypertension while a low percentage presented with diabetes. These findings could be explained by the fact that elderly people are more at risk of hypertension, which results in approximately 50% of ischaemic strokes and significantly increases the risk of haemorrhagic stroke, while diabetes is an independent risk factor to stroke (Banerjee & Paik 2012; World Health Federation 2017). Although the occurrence of HIV and stroke may purely be coincidental, HIV could be an effect modifier to stroke as a result of the associated vasculopathies and cerebrovascular changes (Benjamin et al. 2012). According to Statistics South Africa 2018 general household survey, an estimated 13.1% of South Africa’s population is HIV positive. Unknown HIV data among stroke survivors has been a recurring issue as reported by a previous study conducted by Stone et al. (2020), which found an unknown HIV status in 45% of their study participants. This study could not determine the association between HIV status and LOS because of 49.2% of the participants presenting with an unknown HIV status. The demographic profile and burden of disease profile of the participants will impact the criteria for admission to a stroke unit as well as influence the type of education and counselling conducted on preventing secondary strokes. Understanding the patient profile largely assists in identifying areas for implementation of prevention programmes.

The median LOS for participants in this study was 9 days. In countries such as Ghana and Mozambique, the average was 6 days (Agyemang et al. 2012; Mweshi et al. 2016). In contrast, a study conducted at a Nigerian tertiary hospital showed the average LOS was 13.7 days, when compared with developed countries, the estimated LOS of this study was less than most European countries who report an average of 12.1 days (Somotun et al. 2017). Hospital admission of each patient is costly, and an increase in LOS increases the cost to the hospital. According to a study by Viljoen (2016), the LOS of participants was directly related to the cost to the hospital. The CHBAH accounts department estimated that the financial expenditure for each patient admitted between September 2018 and May 2019 ranged between R3400.40 and R4925.40 per day. Using the data from this study, it was determined that the average cost per admission because of stroke, based on the median (9 days), was between R30603.60 and R44328.60. A delay in rehabilitation services increased the LOS to a median of 13 days, thus increasing the cost per patient admission to R44205.20 – R64030.20. Study participants that were referred for rehabilitation within 3 days resulted in a decreased median LOS (8 days) reducing the cost per patient admission to between R27203.20 and R39403.20. The timely referral to the rehabilitation team decreased the LOS of participants resulting in a reduction in cost to the hospital.

According to the SACSRG (2019), the time from admission to referral for rehabilitation should be between 24 h and 48 h. On average, it took 2 days before participants were referred to the rehabilitation team and 3 days from admission to a first contact with the rehabilitation team. This delay prevents stroke survivors from receiving rehabilitation services early and can lead to secondary complications (Viljoen 2016). The time from admission to initial contact is in line with a study by Chimatiro and Rhoda (2019), which found that on average, in low- to middle-income countries, it took 3 days from admission to referral to PT. The average LOS of participants in this study was 3 days longer than what was found in a previous stroke study at the same facility (Mudzi et al. 2012).

Early rehabilitation intervention has been strongly recommended by the SACSRG (2019), which states that a stroke survivor should be screened for swallowing disorders and mobilised once they are medically stable to decrease the chances of developing deep vein thrombosis, pneumonia, pulmonary emboli and pressure sores as these can increase the LOS and result in poorer functional outcomes or in-hospital death. For such recommendations to be fulfilled, stroke survivors would need to be seen from the first day of admission (SACSRG 2019; Saxena & Prasad 2016). The establishment of a stroke unit would allow stroke survivors to be screened by the rehabilitation team earlier and admitted directly into the unit, reducing the time from admission to referral and initial contact. This improves initiation of early rehabilitation, ongoing discharge planning and allows for effective implementation of stroke care recommendations and multidisciplinary protocols (SACSRG 2019; Canadian Stroke Strategy 2009; Viljoen 2016; Zhu et al. 2009).

In this study, 51.9% of participants required services from all three rehabilitation team departments; therefore, the need for a multidisciplinary approach and dedicated unit to optimise stroke care is reinforced. According to the results, 70% of participants are then referred to continue therapy, either at CHBAH down referral sites or specialised rehabilitation units. Stroke survivors at CHBAH are managed within the general hospital system (Bryer et al. 2010), the involvement of multiple health professionals across many areas results in breakdowns in communication, accountability and teamwork, hindering the success of stroke care provided (Adeloye 2014). As seen from this study, 19.8% of participants passed away in hospital. Research indicates that the implementation of dedicated stroke units reduces the odds of death in this population (Langhorne, Ramachandra & Stroke Unit Trialists’ Collaboration 2020). Given the significant percentage of participants that passed away, it would support the implementation of a dedicated unit to assist in potentially reducing mortality during the course of inpatient admission. An evidence-based and coordinated stroke care pathway through the use of a stroke unit can further reduce the burden of disability and death related to strokes (Platz 2019).

The majority of participants (67.9%) were discharged to resume rehabilitation on an outpatient basis, while only 2.1% were transferred to a specialised rehabilitation hospital. This finding was highlighted in a different study that found over 50% of participants with cerebrovascular disease in South Africa were discharged to resume rehabilitation as an outpatient (Stone et al. 2020). Currently within the region of the research study, participants may be referred to a 15-bed specialised rehabilitation hospital that accommodates patients on a 6–12 week cycle. This would explain why only 2.1% of participants could be referred to a rehabilitation unit as the current infrastructure appear disproportionate to the rehabilitation needs of stroke survivors in the region. This finding highlights the urgency for re-evaluation and establishment of improved infrastructure in an attempt to streamline and provide accessible stroke services in public health.

### Study limitations

The main limitation was missing data as a result of the involvement of multiple field researchers and inconsistent data capturing. Data capturing was affected by lack of training of field researchers as well as the ability to interpret medical records. Missing data were also attributed to insufficient hospital records and unavailable medical investigations. The data capturing was further impacted by delayed confirmation of diagnosis, mixed terminology or unfamiliar abbreviations. There was a significant time limitation for researchers to collect and analyse data because of their daily schedule and clinical tasks.

## Conclusion

The demographic characteristics of the study population was in line with existing literature on stroke populations. There was a higher incidence of strokes in females and participants between the ages of 56 and 76 years, with hypertension being the most common comorbidity. The median LOS of a stroke survivor at CHBAH was 9 days. It was found that a delayed referral to the rehabilitation team resulted in an increased LOS of approximately 5 days and increased the cost per admission from R17002.00 to R24627.00. The care pathway of stroke survivors at CHBAH is not aligned with literature on specialised stroke care in the acute care setting. This results in insufficient time for treatment, impacted patient outcomes and a fragmented team approach.

A dedicated stroke unit or ward could assist in the reduction of time from admission to initial contact with the rehabilitation team, which results in improved patient outcomes and a reduction in LOS (Bryer et al. 2010; SACSRG 2019; Viljoen 2016; Zhu et al. 2009). Reduced LOS of stroke survivors would reduce financial expenditure for public healthcare institutions. The implementation of a stroke unit could lead to a more cohesive management approach by reducing challenges related to discharge planning, communication breakdowns and infrastructure challenges in accordance with various stroke models of care and guidelines published (Bryer et al. 2010; SACSRG 2019; Viljoen 2016; Zhu et al. 2009).

### Implications

This study proposes to contribute to the current body of stroke population demographics and rehabilitation needs within the Johannesburg region. It aims to contribute towards motivating for effective care pathways of stroke survivors while improving patient outcomes and reducing the cost and burden on the healthcare system. The study also suggests the need for further research related to improving stroke care infrastructure across levels of service delivery.
